# Malic Enzyme 1 Is Associated with Tumor Budding in Oral Squamous Cell Carcinomas

**DOI:** 10.3390/ijms21197149

**Published:** 2020-09-28

**Authors:** Chie Nakashima, Tadaaki Kirita, Kazuhiko Yamamoto, Shiori Mori, Yi Luo, Takamitsu Sasaki, Kiyomu Fujii, Hitoshi Ohmori, Isao Kawahara, Takuya Mori, Kei Goto, Shingo Kishi, Rina Fujiwara-Tani, Hiroki Kuniyasu

**Affiliations:** 1 Department of Molecular Pathology, Nara Medical University, 840 Shijo-cho, Kashihara, Nara 634-8521, Japan; c-nakashima@naramed-u.ac.jp (C.N.); shi.m.0310@i.softbank.jp (S.M.); takamitu@fc4.so-net.ne.jp (T.S.); toto1999-dreamtheater2006-sms@nifty.com (K.F.); brahmus73@hotmail.com (H.O.); isao_kawahara@a011.broada.jp (I.K.); pt_mori_t@yahoo.co.jp (T.M.); ilgfgtk@gmail.com (K.G.); nmu6429@yahoo.co.jp (S.K.); rina_fuji@naramed-u.ac.jp (R.F.-T.); 2 Department of Oral and Maxillofacial Surgery, Nara Medical University, 840 Shijo-cho, Kashihara, Nara 634-8522, Japan; kazuyama@naramed-u.ac.jp; 3 Key Laboratory of Neuroregeneration of Jiangsu and Ministry of Education, Co-Innovation Center of Neuroregeneration, Nantong University, Nantong 226001, China; lynantong@hotmail.com

**Keywords:** ME1, tumor budding, EMT, extracellular pH, hypoxia

## Abstract

Budding at the tumor invasive front has been correlated with the malignant properties of many cancers. Malic enzyme 1 (ME1) promotes the Warburg effect in cancer cells and induces epithelial–mesenchymal transition (EMT) in oral squamous cell carcinoma (OSCC). Therefore, we investigated the role of ME1 in tumor budding in OSCC. Tumor budding was measured in 96 human OSCCs by immunostaining for an epithelial marker (AE1/AE3), and its expression was compared with that of ME1. A significant correlation was observed between tumor budding and ME1 expression. The correlation increased with the progression of cancer. In human OSCC cells, lactate secretion decreased when lactate fermentation was suppressed by knockdown of ME1 and lactate dehydrogenase A or inhibition of pyruvate dehydrogenase (PDH) kinase. Furthermore, the extracellular pH increased, and the EMT phenotype was suppressed. In contrast, when oxidative phosphorylation was suppressed by PDH knockdown, lactate secretion increased, extracellular pH decreased, and the EMT phenotype was promoted. Induction of chemical hypoxia in OSCC cells by CoCl_2_ treatment resulted in increased ME1 expression along with HIF1α expression and promotion of the EMT phenotype. Hypoxic conditions also increased matrix metalloproteinases expression and decreased mitochondrial membrane potential, mitochondrial oxidative stress, and extracellular pH. Furthermore, the hypoxic treatment resulted in the activation of Yes-associated protein (YAP), which was abolished by ME1 knockdown. These findings suggest that cancer cells at the tumor front in hypoxic environments increase their lactate secretion by switching their energy metabolism from oxidative phosphorylation to glycolysis owing to ME1 overexpression, decrease in extracellular pH, and YAP activation. These alterations enhance EMT and the subsequent tumor budding. Tumor budding and ME1 expression are thus considered useful markers of OSCC malignancy, and ME1 is expected to be a relevant target for molecular therapy.

## 1. Introduction

Tumor budding refers to a small undifferentiated cancer cell cluster consisting of up to five cancer cells or a solitary cancer cell in the invasive front [1]. These cells are thought to acquire metastatic potential through the acquisition of an epithelial–mesenchymal transition (EMT) phenotype, characterized by decreased cell adhesion and enhancement of stemness [2]. Tumor budding has been shown to correlate with malignant properties in colorectal cancer and has been used as a prognostic marker [3,4]. In oral cancer, tumor budding correlates with lymph node metastasis and worse disease-free survival and overall survival and is considered a prognostic marker [5,6,7].

Hypoxia in advanced cancer microenvironment promotes tumor budding in colorectal cancer [8]. Genes whose expression is induced by hypoxia-inducible factor (HIF)-1α activation by hypoxia promote EMT [9]. In addition, reprogramming of energy metabolism in a hypoxic environment, that is, suppression of oxidative phosphorylation and promotion of glycolysis and lactate fermentation, alter the properties of cancer [10]. In a previous study, we reported that overexpression of malic enzyme (ME)-1 in cancer promotes glutaminolysis, resulting in altered energy metabolism, from oxidative phosphorylation to glycolysis [11]. ME1 decarboxylates malic acid to form pyruvate and ultimately NADPH [12]. In cancer cells, pyruvate produced by ME1 is used for lactate fermentation [13,14]. ME1 expression in cancer cells also leads to energy metabolism reprogramming from oxidative phosphorylation to glycolysis, resulting in decreased oxygen consumption and increased lactate production, promoting tumorigenicity and tumor growth [11,15]. Taken together, the above findings suggest that ME1 might play an important role in tumor budding.

In this study, we investigated the role of ME1 in tumor budding in oral squamous cell carcinoma (OSCC), in which we had found a significant role for ME1 in tumor progression and EMT [11]. The results of this study are expected to clarify the mechanism of tumor budding and lead to the establishment of therapeutic strategies.

## 2. Results

### 2.1. Relationship between Tumor Budding and ME1 Expression in OSCC

**Figure 1:**
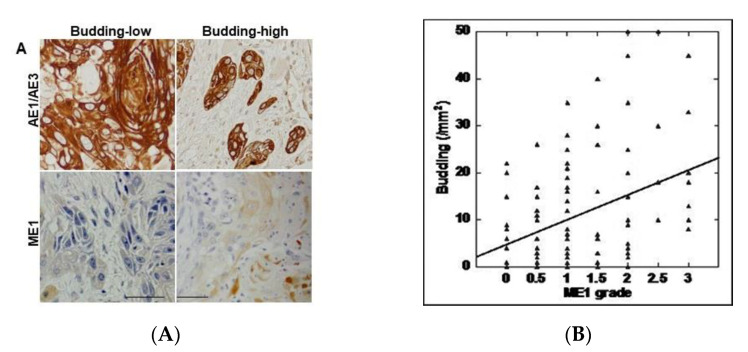
Relationship between tumor budding and malic enzyme)1 expression. (**A**) Tumor budding confirmed by AE1/AE3 immunostaining and ME1 immunoreactivity. Scale bar, 100 µm. (**B**) Correlation between tumor budding and ME1 expression by Spearman regression model. Spearman r = 0.284, *p* < 0.005. ME1, malic enzyme 1; AE1/AE3, cytokeratin AE1/AE3.

To clarify the relationship between tumor budding and ME1 in OSCC, we performed immunostaining of the epithelial marker AE1/AE3 and of ME1 in 96 specimens of OSCC (Figure 1A and Table 1). As shown in Figure 1, ME1 expression was low in cases where budding was not seen at the tumor front, whereas it was higher in cases where budding was observed at the tumor front. As shown in Table 2, tumor budding and ME1 expression were significantly correlated with cancer progression according to both pT and pStage classifications. However, a significant correlation was observed only with ME1 with respect to grade and only with budding in the presence of lymph node metastasis.

Next, the correlation between the number of budding cells and ME1 expression was examined (Table 2). In all cases, a significant correlation was found between the two (*p* = 0.005) (Figure 1B). Furthermore, when the correlation between tumor budding and ME1 expression was compared with T factor, N factor, and stage, a stronger correlation was observed in cases that progressed with respect to all factors. This suggests that tumor budding and ME1 are deeply involved in the progression of OSCC.

### 2.2. Effect of ME1 on Extracellular pH

**Figure 2:**
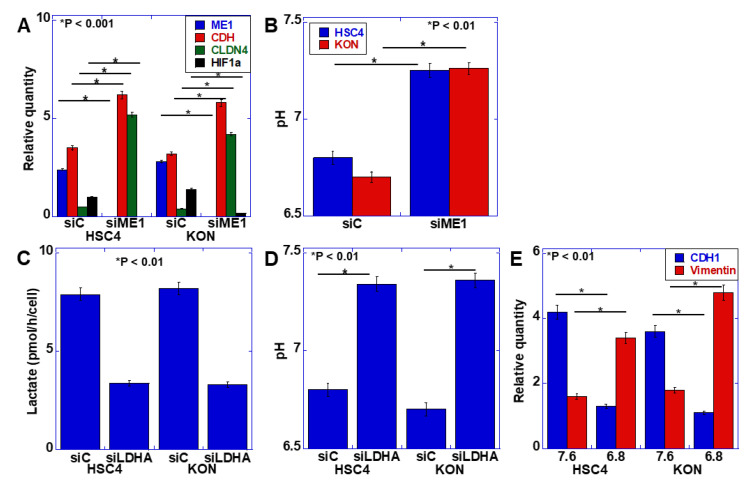
Effect of extracellular pH on the epithelial–mesenchymal transition (EMT) phenotype in HSC4 and KON oral squamous cell carcinoma (OSCC) cells. (**A**,**B**) Effect of the knockdown of ME1 on mRNA expression of EMT-associated genes measured by quantitative PCR (**A**) and extracellular pH (**B**) in HSC4 and KON cells. (**C**,**D**) Effect of the knockdown of lactate dehydrogenase A (LDHA) on extracellular lactate concentration (**C**) and pH (**D**) in HSC4 and KON cells. (**E**) Effect of medium pH on the EMT phenotype in HSC4 and KON cells. Expression was measured by quantitative PCR. Error bar, standard deviation tested by Student’s *t*-test in three independent trials. CDH, E-cadherin; CLDN, claudin; HIF, hypoxia-inducible factor; siRNA, small interference RNA; siME, siRNA for ME1; siC, control siRNA; siLDHA, siRNA for LDHA.

The EMT phenotype has been identified as a malignant characteristic of budding cancer cells [2]. Our previous studies revealed that ME1 promotes the Warburg effect and is involved in the induction of EMT [11]. Therefore, we examined the relationship between the Warburg effect and EMT. First, ME1 was knocked down in the human oral SCC cell line HSC4, and the expression of EMT-related factors was examined (Figure 2A). Knockdown of ME1 increased the expression of E-cadherin and claudin (CLDN)-4 and decreased the expression of HIF-1α. Furthermore, the possible alteration in extracellular pH was examined by considering the effect of ME1 on the Warburg effect (Figure 2B). Knockdown of ME1 altered the acidic extracellular pH to neutral. Lactate dehydrogenase A (LDHA) was then knocked down to confirm that the alteration in the extracellular pH was due to changes in glycolysis–lactate fermentation (Figure 2C,D). Consequently, lactate level in the culture solution decreased, and the pH increased. To investigate the effect of extracellular pH on EMT, the pH of the culture solution was adjusted to 7.6 and 6.8, and HSC4 cells were cultured (Figure 2E). In the acidic culture medium, E-cadherin expression was decreased, and vimentin expression was increased, which indicated that the EMT phenotype was induced in the acidic culture medium compared with the neutral culture medium.

### 2.3. Relationship between the Warburg Effect and Lactate Secretion

**Figure 3:**
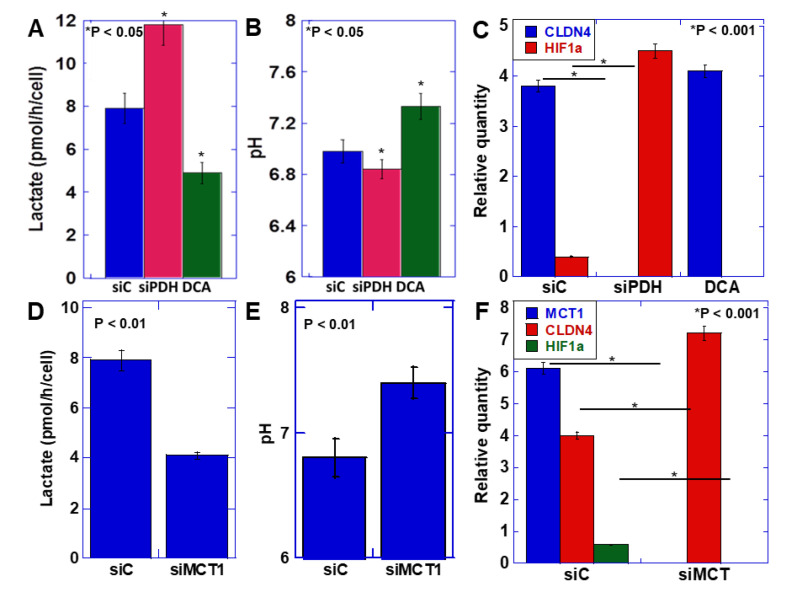
Effect of lactate on the EMT phenotype in HSC4 OSCC cells. (**A**–**C**) Effect of knockdown of pyruvate dehydrogenase (PDH) or inhibition of PDH kinase by dichloroacetate (DCA) on extracellular lactate (**A**), extracellular pH (**B**), and mRNA expression of EMT-associated genes measured by quantitative PCR (**C**) in HSC4 cells. (**D**–**F**) Effect of knockdown of monocarbonate transporter (MCT)1 on extracellular lactate (**D**), extracellular pH (**E**), and mRNA expression of EMT-associated genes measured by quantitative PCR (**F**) in HSC4 cells. Error bar, standard deviation tested by Student’s *t*-test in three independent trials. siPDH, siRNA for PDH; siMCT1, siRNA for MCT1.

EMT was induced by the acidification of the extracellular pH, suggesting that the transition promoted by ME1 from oxidative phosphorylation of energy production to glycolysis and lactate fermentation is important for EMT acquisition. Therefore, we next knocked down pyruvate dehydrogenase (PDH), which induces oxidative phosphorylation through metabolism of pyruvate, the final metabolite of glycolysis, or inhibited PDH kinase, which induces glycolysis by dichloroacetate (DCA) (Figure 3A–C). Knockdown of PDH increased lactate production and decreased the extracellular pH (Figure 3A). In contrast, inhibition of PDH kinase decreased lactate production and increased the extracellular pH (Figure 3B). On examining the expression of CLDN4 and HIF1α, which are involved in the EMT phenotype (Figure 3C), CLDN4 expression was found to be decreased, and HIF1α expression was enhanced by PDH knockdown, indicating promotion of the EMT phenotype. In contrast, inhibition of PDH kinase increased CLDN4 expression and decreased HIF1α expression, indicating suppression of the EMT phenotype.

Next, to confirm the significance of lactate secretion compared with the increase in intracellular lactate levels, the monocarbonate transporter (MCT)-1, which promotes secretion of lactate extracellularly, was knocked down. (Figure 3D–F). As a consequence, the extracellular lactate level was found to decrease, and the extracellular pH increased. At this time, CLDN4 expression increased, and HIF1α expression decreased, indicating suppression of the EMT phenotype. As observed above, the decrease in extracellular pH due to the increase in lactate secretion accompanying the enhancement of the Warburg effect induced the EMT phenotype.

### 2.4. Effects of Hypoxic Environment on Cancer Cells

**Figure 4:**
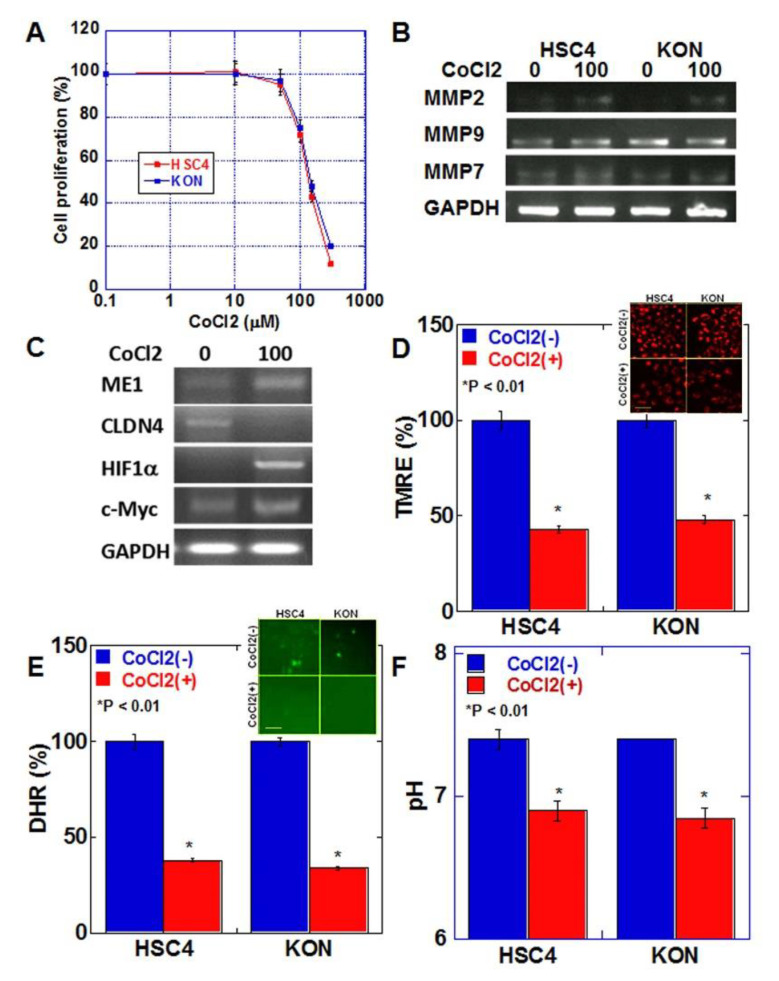
Effect of hypoxia on EMT and mitochondrial function in HSC4 and KON OSCC cells. (**A**) Effect of CoCl_2_-induced hypoxia on cell proliferation in HSC4 and KON OSCC cells. (**B**) Effect of hypoxia on mRNA expression of MMPs in HSC4 and KON OSCC cells. (**C**) Effect of hypoxia on mRNA expression of EMT-associated genes. (**D**–**F**) Effect of hypoxia on mitochondrial membrane potential (TMRE assay). (**D**), mitochondrial reactive oxygen species (ROS, DHR assay) (**E**), and extracellular pH (**F**). Inserts in (**D**,**E**) show fluorescent images. Error bar, standard deviation tested by Student’s *t*-test in three independent trials. Scale bar, 50 μm. GAPDH, glyceraldehyde 3-phosphate dehydrogenase; TMRE, tetramethylrhodamine ethyl ester; ROS, reactive oxygen species; DHR123, dihydrorhodamine 123.

We next examined the effect of hypoxic environment, which is characteristic of budding cancers, on cancer cells [17] (Figure 4). Cell proliferation was examined after treating both oral SCC cell lines with various concentrations of CoCl_2_ to induce a chemical hypoxic environment (Figure 4A). In both cell lines, growth inhibition of 20–30% was observed at 100 μM of CoCl_2_. In HSC4 and KON cells, treatment with 100 μM CoCl_2_ increased the expression of matrix metalloproteinase (MMP)9 and MMP7 but not of MMP2 (Figure 4B). CoCl_2_ treatment increased the expression of ME1 and HIF1α and decreased the expression of CLDN4 in HSC4 cells (Figure 4C). The expression of c-Myc, which promotes glycolysis, was also enhanced. In contrast, in the hypoxic environment, mitochondrial membrane potential, extracellular pH, and mitochondrial reactive oxygen species (ROS) levels were decreased in both cell lines (Figure 4D–F).

### 2.5. Yes-Associated Protein (YAP) Activation by ME1

**Figure 5:**
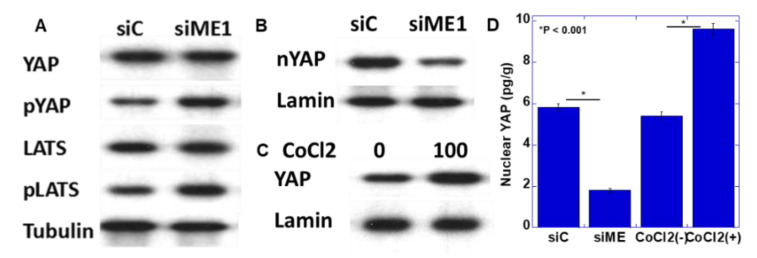
Effect of ME1 and hypoxia on yes-associated protein (YAP) activation in HSC4 OSCC cells. (**A**,**B**) Effect of knockdown of ME1 on phosphorylation (**A**) and nuclear translocation (**B**) of YAP in HSC4 cells. (**C**) Effect of CoCl_2_-induced hypoxia on nuclear translocation of YAP in HSC4 cells. (**D**) Concentrations of nuclear YAP were measured by ELISA. Tubulin and lamin were examined as loading controls. pYAP, phosphorylated YAP; LATS, large tumor suppressor; pLATS, phosphorylated LATS; siME, siRNA for ME1.

In a previous study, we reported that YAP activation induces the EMT phenotype in oral SCC [18]. Therefore, YAP activation as the EMT induction mechanism by ME1 was examined by knocking down ME1 (Figure 5A,B). Knockdown of ME1 increased the phosphorylation levels of YAP and large tumor suppressor (LATS) and decreased nuclear YAP levels. This suggested that YAP is activated by ME1. In addition, hypoxic treatment increased nuclear YAP levels (Figure 5C).

## 3. Discussion

In the present study, budding at the invasive front of cancer was found to be closely related to cancer progression. It has been suggested that a hypoxic environment, promotion of the Warburg effect, decrease in extracellular pH, and EMT are responsible for tumor budding and acquisition of the malignant phenotype. Furthermore, ME1 plays an important role in this process.

At the invasive front, cancer cells tend to experience hypoxia because they invade the stromal tissue with low blood vessel density. In oral melanoma, it has been reported that hypoxia is seen at the invasive front, and hypoxia responses such as HIF1α and vascular endothelial growth factor (VEGF) upregulation have been observed [17]. Our study also showed that alterations in HIF1α and ME1 expression occurred concomitantly, suggesting a relationship between tumor budding and hypoxia. Furthermore, it was revealed that the expression of ME1 was induced by hypoxia. A previous study reported that ME1 expression is lowered by HIF1α knockdown [19]. These findings suggest that ME1 is an HIF1α target gene, and ME1 overexpression at the invasive front can be considered as a hypoxic response.

In a hypoxic environment, expression of MMP9 and MMP7 is upregulated by HIF1α activation [20,21]. Our data showed increase of MMP9 and MMP7 in hypoxic conditions, which suggests increase of invasion ability. VEGF, whose secretion is induced by hypoxia, promotes angiogenesis and lymphangiogenesis, facilitating the metastatic spread of cancer cells [22]. Hypoxia activates Jagged2 and promotes stemness and EMT via NOTCH and AKT signals [23]. Furthermore, hypoxic cancers are more invasive and have a poorer prognosis than non-hypoxic cancers [24]. Thus, hypoxia is deeply associated with the promotion of the tumor budding phenotype.

Since hypoxia is strongly involved in tumor budding, we examined the energy metabolism associated with the hypoxic environment and found that ME1 expression and lactate levels were increased. Liao et al. reported that ME1 expression in cancer cells leads to energy metabolism reprogramming from oxidative phosphorylation to glycolysis, resulting in decreased oxygen consumption, increased lactate production, and subsequent promotion of tumorigenicity and tumor growth [11,15]. According to our data, hypoxia decreased the mitochondrial membrane potential, suppressed oxidative phosphorylation, and decreased oxidative stress. A correlation between budding at the invasive front and promotion of glycolysis via increased GLUT1 expression, which enhances glucose intake, has been previously reported [25]. Fructose-1,6-biophosphase promotes oxidative phosphorylation but is suppressed by EMT-inducing Snail, resulting in a switch in the glycolytic pathway of energy metabolism during the EMT process [26]. Thus, a mutual promotive relationship is suggested between energy metabolism reprogramming and EMT phenotype at the tumor invasive front.

Furthermore, in this study, lactate secretion increased and extracellular pH decreased as a result of promotion of the Warburg effect. The decrease in extracellular pH was strongly correlated with EMT. A decrease in extracellular pH promotes growth, migration, invasion, and metastasis of cancer cells in an acidic environment via GPR4, GPR65 (TDAG8), GPR68 (OGR1), and GPR132 (G2A), which are pH-sensing G protein-coupled receptors (GPCRs) [27].

An acidic extracellular pH also suppresses immune cells and reduces responsiveness to anti-cancer drugs [27]. We have also reported that impairment of the barrier function of tight junctions by an anti-CLDN4 antibody raises the pH of the tumor microenvironment and reduces the stemness of cancer cells [28]. In addition, a low pH in the cancer cell microenvironment promotes infiltration of myeloid-derived suppressor cells, regulatory T cells, and tumor-associated macrophages and induces PD-L1 expression in cancer cells, which impairs T-cell antitumor immunity [29]. Acidification of the cancer microenvironment is thought to be caused by increased lactate secretion. In our study, the inhibition of pyruvate metabolization to acetyl-CoA by PDH knockdown increased lactate production and decreased the extracellular pH. Conversely, LDHA knockdown and PDH activation by PDH kinase inhibition reduced lactate and increased the extracellular pH. Furthermore, the extracellular pH was increased by knocking down MCT1, which is a transporter that secretes lactate. Extracellular carbonic anhydrases and monocarboxylate transporters are also known to play an important role in inducing a low extracellular pH under hypoxia, which promotes invasion, metastasis, and stemness of cancer cells [29].

In our study, YAP was activated in a hypoxic environment and was abrogated by knockdown of ME1. We already shoed that YAP activation is a significant factor that promotes the EMT phenotype and is deeply involved in the progression of oral SCC [18]. In hypoxia, YAP is activated through upregulation of GPRC5A by HIF1α [30]. Reciprocally, activated YAP stabilizes HIF1α and enhances its action [31]. In addition, YAP is activated by enhanced glycolysis–lactate fermentation [32]. The inhibition of YAP activation by ME1 knockdown observed in our study is considered to be associated with the suppression of ME1 energy metabolism reprogramming. Thus, YAP is thought to induce EMT in response to hypoxia and energy metabolism reprogramming.

As described above, reprogramming of the energy metabolism pathway from oxidative phosphorylation to glycolysis–lactate fermentation, increased lactate secretion, and consequent YAP activation were closely involved in the induction of the EMT phenotype. Furthermore, the extracellular pH increased due to ME1 knockdown, suggesting that ME1 is strongly involved in energy metabolism reprogramming.

## 4. Materials and Methods

### 4.1. Tissue Arrays

Tissue array slides of OSCCs (96 cases) were obtained from US Biomax Inc. (Rockville, MD, USA). Duplet slides were immunostained with anti-ME1 and anti-AE1/AE3 antibodies (Abnova Corporation, Taipei City, Taiwan), and the association between clinicopathological parameters and ME1 expression levels was determined.

### 4.2. Immunohistochemistry

Formalin-fixed, paraffin-embedded surgical specimens were cut into 4 μm-thick sections. Consecutive 4 μm sections were immunohistochemically stained using the immunoperoxidase technique described previously [33]. The sections were incubated with 0.5 µg/mL anti-ME1 or anti-AE1/AE3 antibodies for 2.5 h at room temperature. Secondary antibodies (Dako, Carpinteria, CA, USA) were applied for 1 h. Tissue sections were color-developed with diaminobenzidine hydrochloride (Dako) and counterstained with Meyer’s hematoxylin (Sigma Chemical Co., St. Louis, MO, USA). ME1 immunoreactivity was classified according to our previous study [11]. Grade 0 indicates no expression, Grade 1 indicates an expression equivalent to that in normal oral squamous epithelium, Grade 2 is an intermediate expression, and Grade 3 indicates strong expression [34].

### 4.3. Human OSCC Cell Lines

We purchased HSC4 and KON human OSCC cell lines from Dainihon Pharmaceutical Co. (Tokyo, Japan). Cells were cultured and maintained in Dulbecco’s modified Eagle’s medium (DMEM) supplemented with 10% fetal bovine serum (FBS) at 37 °C in 5% CO_2_. Cell growth was assessed using the 3-(4,5-dimethylthiazol-2-yl)-5-(3-carboxymethoxyphenyl)-2-(4-sulfophenyl)-2H-tetrazolium (MTS) assay, as previously described [33]. Culture media with pH 7.6 and pH 6.9 were prepared by adjusting the pH with EDTA and HCl (WAKO), respectively, from regular DMEM. CoCl_2_ and DCA were purchased from WAKO Pharmaceutical Co. Ltd. (Osaka, Japan).

### 4.4. Extracellular pH

Cells (1 × 10^4^) were cultured with 2 mL of DMEM for 48 h. The pH of the culture medium was measured with a pH meter (Chemical Instruments, Co. Ltd., Hachioji, Japan).

### 4.5. Small Interfering RNA

Stealth Select RNAi (siRNA) for human ME1, LDHA, PDH, and MCT1 was purchased from Santa Cruz Biotechnology. AllStars Negative Control siRNA was used as a control (Qiagen, Venlo, The Netherlands). The cells were transfected with 20 nM siRNA using Lipofectamine 2000 (Invitrogen Corp.; Carlsbad, CA, USA) according to the manufacturer’s recommendations.

### 4.6. Reverse Transcription Polymerase Chain Reaction (PCR)

Total RNA (1 μg) was used to synthesize cDNA using a ReverTra Ace quantitative PCR (qPCR) RT kit (Toyobo, Osaka, Japan). PCR was performed as specified by the manufacturer. PCR products were electrophoresed on 2% agarose gels and visualized using ethidium bromide. The primer sets are listed in Table 3. Primers were synthesized by Sigma-Genosys (Ishikari, Japan).

### 4.7. Protein Extraction

To prepare whole-cell lysates, oral SCC cells (1 × 10^7^) were washed twice with cold PBS, harvested, and lysed with 0.1% SDS-containing RIPA buffer (Thermo Fisher Scientific, Tokyo, Japan) [34]. Cell fractions were extracted using a Cell Fractionation Kit (Abcam, Cambridge, MA, USA), according to the manufacturer’s instructions [35]. Protein assays were performed using a Protein Assay Rapid Kit (Wako Pure Chemical Corporation, Osaka, Japan).

### 4.8. Immunoblot Analysis

Whole-cell lysates of HSC4 cells were prepared as previously described [34]. Lysates (20 μg) were separated by immunoblot analysis using 12.5% sodium dodecyl sulfate polyacrylamide gel electrophoresis (SDS-PAGE), followed by electrotransfer onto nitrocellulose filters. The filters were incubated with primary antibodies, followed by secondary peroxidase-conjugated IgG antibodies (Medical and Biological Laboratories). An anti-tubulin antibody was used for loading control (Oncogene Research Products, Cambridge, MA, USA). The immune complexes were visualized using an Enhanced Chemiluminescence Western-blot detection system (Amersham, Aylesbury, UK). Antibodies against YAP1, phosphorylated YAP1 (pS127), GAPDH (glyceraldehyde-3-phosphate dehydrogenase), large tumor suppressor kinase 1 (LATS1), phosphorylated LATS (pThr1079) (Cell Signaling Technology, Beverly, MA, USA), tubulin (Zymed Laboratories Inc., South San Francisco, CA, USA), and lamin (Proteintech Group Inc., Rosemont, IL, USA) were used as primary antibodies.

### 4.9. Mitochondrial Staining

Mitochondria were stained with tetramethyl rhodamine (TMRE) and dihydrorhodamine 123 (DHR) at 37 °C for 24 h to assess mitochondrial membrane voltage (Takara Bio Inc., Kusatsu, Japan) and observed via fluorescence microscopy (Keyence, Osaka, Japan).

### 4.10. Determination of Concentrations of Lactate and Nucelar YAP

Lactate concentrations were determined using a D-Lactate Assay kit (Cayman Chemical, Ann Arbor, MI, USA). YAP concentrations were determined using a human YAP1 ELISA kit (Abcam). The assays were performed according to the manufacturers’ instructions.

### 4.11. Statistical Analysis

Statistical significance was assessed using the χ^2^-test and Student’s *t*-test with the assumption of Gaussian distribution according to the Kolmogorov and Smirnov method. Linear regression was evaluated with Spearman’s r. Analyses were performed using InStat software (GraphPad, Los Angeles, CA, USA). Statistical significance was defined as *p* < 0.05.

## 5. Conclusions

**Figure 6:**
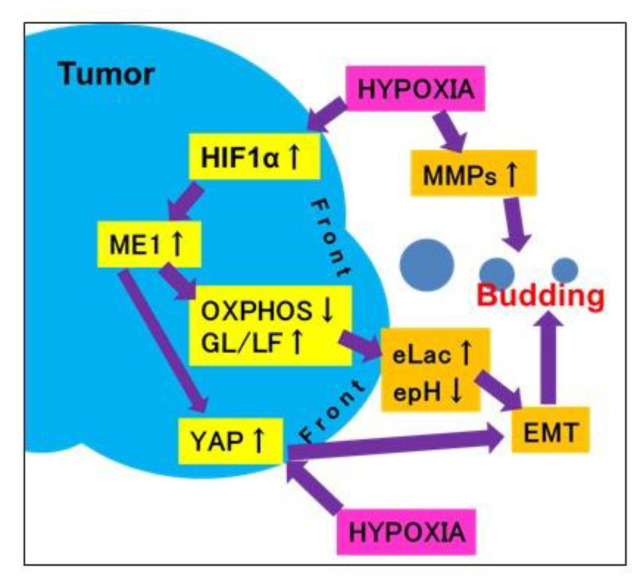
Schema of the mechanism of tumor budding. When cancer cells at the invasive front suffer hypoxia, they overexpress ME1, which leads to reprogramming of the energy metabolism from oxidative phosphorylation to glycolysis/lactate fermentation. This subsequently increases lactate secretion and decreases the extracellular pH and YAP activation. Due to these alterations, cancer cells at the invasive front undergo budding via an increase in stemness, invasion ability, and EMT phenotype. epH, extracellular pH; eLac, extracellular lactate; OXPHOS, oxidative phosphorylation; MMP, matrix metalloproteinase.

As shown in the schematic illustration (Figure 6), in cancer cells at the tumor invasive front, a hypoxic environment increases lactate secretion due to switching of the energy metabolism from oxidative phosphorylation to glycolysis following ME1 overexpression and decreases extracellular pH and YAP activation. These alterations promote tumor budding by enhancing EMT of cancer cells with stem cell properties and invasion ability. Thus, tumor budding is a marker of the malignant phenotype of cancer; it is considered that budding cancer cells themselves have acquired a malignant phenotype. In addition, in our study, the correlation between tumor budding and ME1 expression increased with the progression of cancer, suggesting that ME1 strongly promotes tumor budding and the malignant phenotype in OSCCs. We previously reported the effectiveness of inhibition of ME1 using lantanid [11]; however, this strategy has issues related to lantanid’s derivatives and stability. In the future, the discovery of a more useful ME1 inhibitor is desired for targeting tumor budding.

## Figures and Tables

**Table 1:** Relationship between ME1 expression or tumor budding and clinicopathological parameters in 96 oral squamous cell carcinomas.

Parameter	Case Number	ME1 Expression	*p* *	Budding (/mm^2^)	*p* *
Age (years)					
≤59	59	1.28 ± 0.81		14.2 ± 15.1	
≤60	37	1.12 ± 0.89	NS	10.0 ± 14.1	NS
Sex					
F	14	1.29 ± 0.96		10.8 ± 8.2	
M	82	1.21 ± 0083	NS	12.9 ± 15.7	NS
Site					
Gingiva	26	1.19 ± 0.71		15.6 ± 17.5	
Tongue	35	1.41 ± 0.94		13.1 ± 10.0	
Pharynx	17	1.09 ± 0.69		12.1 ± 16.1	
Floor	18	1.56 ± 1.00	NS	11.5 ± 18.1	NS
Grade					
1	38	1.5 ± 0.8		12.5 ± 15.4	
2	29	1.3 ± 0.9		13.8 ± 16.4	
3	29	0.8 ± 0.7	0.0199	11.6 ± 12.3	NS
pT					
1	13	0.9 ± 1.0		2.8 ± 6.2	
2	37	1.3 ± 0.8		5.1 ± 4.4	
3	39	1.2 ± 0.8		19.4 ± 11.1	
4	7	2.1 ± 0.3	0.0175	22.1 ± 24.8	<0.0001
pN					
0	57	1.3 ± 0.9		10.9 ± 12.1	
1	28	1.1 ± 0.7		14.9 ± 13.9	
2	11	1.4 ± 0.8	NS	18.8 ± 11.5	<0.0001
pStage					
1	7	1.0 ± 1.2		1.6 ± 2.9	
2	22	1.4 ± 0.9		3.7 ± 4.4	
3	60	1.1 ± 0.8		14.8 ± 11.3	
4	7	2.1 ± 0.3	0.0156	22.1 ± 24.8	<0.0001

Tumor progression, stage, and histological grades were determined according to the Union for International Cancer Control TNM classification system [16]. NS, not significant. * *p* value is calculated by Studen’s *t*-test.

**Table 2:** Correlation between ME1 expression and tumor budding.

Parameters	Case Number	Spearman r	*p* *
All cases	96	0.284	0.005
pT1–2	50	0.3633	0.0103
pT3–4	46	0.4783	0.0004
pN0	57	0.3085	0.0132
pN1–2	39	0.5624	0.0016
pStage1–2	29	0.3023	0.0129
pStage3–4	67	0.5599	<0.0001

Tumor progression and stage were determined according to the Union for International Cancer Control TNM classification system [16]. * *p* value is calculated by Spearman r regression test.

**Table 3:** Primer sets for RT-PCR.

Gene Name	Gene Bank ID	Upper/Lower	Primer Sequence
ME1	NM_002395.5	U	GGATTGCACACCTGATTGTG
		L	TCTTCATGTTCATGGGCAAA
CDH	Z13009.1	U	TGCCCAGAAAATGAAAAAGG
		L	GTGTATGTGGCAATGCGTTC
CLDN4	NM_001305.4	U	CTCCATGGGGCTACAGGTAA
		L	AGCAGCGAGTCGTACACCTT
HIF1α	AF208487.1	U	GAAAGCGCAAGTCCTCAAAG
		L	TGGGTAGGAGATGGAGATGC
MCT1	AY364258.1	U	TCCTTTTATCCTGCCACACC
		L	GCATGCTGTTTTCCTTCTGC
MMP2	KR710613.1	U	ATGACAGCTGCACCACTGAG
		L	ATTTGTTGCCCAGGAAAGTG
MMP9	NM_004994.3	U	TTGACAGCGACAAGAAGTGG
		L	GCCATTCACGTCGTCCTTAT
MMP7	NM_002423.5	U	GAGTGCCAGATGTTGCAGAA
		L	AAATGCAGGGGGATCTCTTT
c-Myc	NM_002467.4	U	TTCGGGTAGTGGAAAACCAG
		L	CAGCAGCTCGAATTTCTTCC
*GAPDH*	BC025925.1	U	GAGTCAACGGATTTGGTCGT
		L	TTGATTTTGGAGGGATCTCG

## Abbreviations

EMT	epithelial–mesenchymal transition
HIF	hypoxia-inducible factor
ME	malic enzyme
OSCC	oral squamous cell carcinoma
CLDN	claudin
LDHA	lactate dehydrogenase A
PDH	pyruvate dehydrogenase
DCA	dichloroacetate
GPCR	G protein-coupled receptor
